# Hints Towards an Inquiry, Whether Tubercular Tendency May Be Eradicated from the System, and by What Means

**Published:** 1857-09

**Authors:** B. Woodward

**Affiliations:** Galesburg, Illinois


ARTICLE II.—*Hints, towards an enquiry, whether Tubercular tendency may be eradicated from the system, and by what means.*—By B. WOODWARD, M. D., Galesburg, Illinois, 1857.

That tubercular disease is, in all its essentials, of scrofulous origin, is the general opinion of the profession. That it is hereditary is also acknowledged. What this hereditary tendency is, or in what it consists, is not fully answered. Paget in his Surgical Pathology, page 33, says, "It seems to be established as a general law in pathology, that diseased structures evidently incorporate materials that *had their origin, or previous existence* in the blood. Such are most of those inoculable, or other blood diseases in which morbid organisms are produced." Carpenter, Human Physiology, page 335, assumes as a fact that there is a constitutional state of abnormal nutrition which causes strumous and tubercular disease; and in a note to page 254, says, "The author is much disposed to agree with Dr. Handfield Jones in believing that a chronic fibroid degeneration resulting from the substitution of a lowly organized fibrous tissue for the proper texture of the part, may take place like tubercular generation, without the occurrence of inflammation properly so called." He thus recognises a constitutional cause, in opposition to those pathologists who regard inflammation as the only cause of tubercle. Wood, vol. 1, pages 114 and 115, says, "That in the present state of our knowledge the question, what is the nature of this affection denominated tubercular disease, and of which tubercle is a mere incident, cannot be fully answered." But he ascribes it to a "condition of the blood," and says, "we are thrown back upon some original vice of the constitution." After enumerating a number of causes which favor the development of tubercle, he goes on to say, that "there are many individuals upon whom all these causes may be made to operate, and so intensely as to produce death, without giving rise to this particular disease." His train of reasoning is remarkably lucid and satisfactory.

Can this "original vice," "taint," "poison" or "disposition be so eradicated, as either to prevent, or greatly lessen the probability of its hereditary transmission, and prevent to a great degree its development in those in whom this predisposition exists? To satisfactorily solve these problems, requires a more thorough knowledge of therapeutics and pathology than I can lay claim to; but, believing that God has never permitted any evil, either physical or moral, to exist without a corresponding remedy, I am led to the belief, that even the tubercular diathesis may be eradicated. I must so far beg the question as to take for granted, that the strumous and tubercular diatheses are essentially one, having different manifestations, and that they are caused by a taint or poison in the system. Paget, Surg. Pathology, page 669, has a long and lucid article on this subject, in which he doubts the actual identity of scrofula and tubercular disease, yet admits that the tubercular form is generally dependent on the strumous diathesis. His article being long, I will not attempt to quote his words, but beg of those interested in the question, to examine his article carefully, and see the general bearing it has on the subject now under consideration. Paget, Wood, Horner and almost all other writers consider tubercular disease as one of debility. Paget, page 667, says "this general state of the constitution, whether natural, or artificially generated, is fairly expressed by such terms as "delicacy of constitution, general debility, defective vital power, irritability without strength." We know that the red corpuscles of the blood are notably diminished, and emaciation to a greater or less degree is a prominent symptom. If then, the disease be one of debility, and having for its origin an occult poison; we are prepared for the questions—can it be eradicated? and by what means? Certain diseases are caused by a poison taken into the system. This poison is material, that it has individuality, and is not a mere state there can be no doubt. It is propagated by contact, by inhalation, which it could not be if it were a state or condition. So must the poison of tubercular disease be an entity. It is a something propagating itself—by all if you please.

I cannot conceive of an entity, existing pathologically, and not physiologically, without at the same time conceiving of the possibility of its being destroyed and removed from the system. It is on the action of certain agents introduced into the system, in some cases so modifying the constitution as to make it incapable of being acted on by other agents, (as the vaccine virus) and in other cases neutralising poisons already existing in the system, and causing them to be eliminated—that I base the belief, that the tubercular tendency, may be so overcome in the young, and in those in whom it has not been fully developed, that in the one case it shall not be transmittable, and in the other, that its full development shall be prevented. Certain agents act on certain poisons as neutralisers. In struma and secondary syphilis, iodine acts as a specific, if I may be allowed to use the term; and if its use is persevered in, the poison is eradicated. Mercury is an antidote to syphilitic poison in its primary condition. Quinine is an antidote for the poison of intermittents. How these agents act, is beyond our present knowledge. Iodine and mercury, so far as we can understand, must act on the poison itself, or as catalytics. Quinine may act in the same way, or it may act as a restorative, in the same way that iron does.

There are those who hold, that tuberculosis depends on a faulty formation ; and point to the narrow and generally faulty form of the thorax of those thus predisposed. But I cannot agree with them, and am led to look on this faulty formation only as an *evidence* of the *predisposition*. The tubercular tendency cannot always be detected by form alone, however close the inspection, though it may be generally diagnosed by form, feature, color and texture of skin, eyes, hair, nails, etc.

In attempting to eradicate tubercular disease we have three indications for treatment. First, to neutralise and overcome the poison, or taint. Second, to expand the chest and restore the thorax to a healthy form. Third, to overcome the general debility, and give the requisite tonicity to the system: to these must be added a fourth, in those whom the disease has been developed, viz: to remove the morbid product or tubercular deposit. How shall we meet them? As this article is only meant to be suggestive, I will neither occupy your pages, or tax the patience of your readers, by more than a glance at this part of the subject.

If we admit the doctrine that "Strumous and tubercular diseases are either identical or nearly allied," we must be led to the belief, that the same class of medicines which are known to be efficacious in the one, will be also in the other. Those designed to meet the first indication, or remove the poison, must of course be hæmatics, and these again must be catalytics, for there is a special catalytic action for them to perform.—Allow me here to refer to "Headland on the action of medicines" article hæmatics, pages 191, 196, 199 and 211, for the arguments to prove that all hope of eradicating the tubercular poison, must rest on the action of catalytics. His article does not refer particularly to tubercular disease, but is on the *action* of catalytics. If his doctrines are true (and they are supported by sound reasoning and experience,) they cover this whole ground. In struma, iodine is the remedy most relied on, and its catalytic action is in this case its sole operation, and we are led to the conclusion that this and other agents, having the same ac-

26

tion, must, if any will, be beneficial in tubercular disease. The faults of form must be met by all those hygienic measures which tend to expand the thorax and give power to the respiratory muscles. The debility is to be overcome by exercise, pure air, nourishing diet and tonics, particularly chloride of sodium and iron. Here allow me to refer your readers to an article of mine which you did me the honor to publish in your journal for June last. The consideration of the fourth indication, or the removal of the morbid product, in the developed cases, I shall at present pass over.

I suggest to the profession, whether it is not possible to treat children and young persons, by such remedial and hygienic measures, as shall eradicate this tubercular tendency, whether it be hereditary or acquired, and lay the foundation for vigorous health? Cannot the causes of almost of universal prevalence consumption be removed? Physicians are the true conservators of the public health, and it is for them to look at these questions in all their bearings.

Schools, as they now exist, are the most fruitful nurseries of consumption. Crowded, stove-warmed, illy ventilated rooms, necessitate the breathing an atmosphere deprived of the most of its oxygen and loaded with effluvium, irritating the lungs and oppressing the brain. Cramped and uneasy positions while sitting, and lounging, careless attitudes while standing, combine to distort the spine and contract the thorax. To all this we must add the over stimulation of the brain and nervous system so prevalent in our higher schools, especially for females. Here we have a combination of causes sufficient to develop tubercular disease in every one having the least tendency to it. In Dr. Meigs' work on "Woman and her Diseases," in his celebrated conversation with "Miss Helen Blanque" the want of proper exercise and air is admirably pointed out; on page 367, he gives his own views, and those of Miss Catharine Beecher on the physical education of girls, and on page 377 will be found a most instructive article on the effect which an over mental stimulation will have on their health. True, Dr. Meigs is not speaking particularly of tubercular disease, but of the controlling effects over menstruation, yet we know that one of the most fruitful sources of tuberculosis, is an abnormal arrest of the menstrua.

The same holds good in many instances with boys and young men. Look at the mass of students in our colleges, and particularly in our theological seminaries, and mark the proportion who give unmistakeable evidence of tubercular disease, and say to what else it can be ascribed, but the unhealthy and unnatural physical and mental training to which they are subjected. Are these things without a remedy? Is it not practicable to establish schools on such a system that they shall be under the supervision of medical men of high standing; and cannot schools be established in or near all our large cities, where those children may be placed who may be predisposed to consumption, educated, and at the same time treated for the vice of their constitutions? Such schools under the care of competent physicians would command the confidence of the public, and ensure that true, just, and judicious measures would be adopted. I am convinced that if such measures were adopted we should soon see their beneficial results. Will not our medical men give this subject the examination which its importance demands, and give us their views through the columns of your journal? It will not do to say, "It is visionary and cannot be accomplished." True it is as yet only theoretical, but *if the theory is correct, it is practicable*, for every truth can, and must be made available in human progress.

If this article should have the effect of drawing the attention of my medical brethren to the subject, I shall be satisfied, though they should demolish every position I have taken, believing as I do, that the truth and the right will eventually prevail.

